# Cardiometabolic phenotypes and mitochondrial DNA copy number in two cohorts of UK women

**DOI:** 10.1016/j.mito.2017.08.007

**Published:** 2018-03

**Authors:** Anna L. Guyatt, Kimberley Burrows, Philip A.I. Guthrie, Sue Ring, Wendy McArdle, Ian N.M. Day, Raimondo Ascione, Debbie A. Lawlor, Tom R. Gaunt, Santiago Rodriguez

**Affiliations:** a MRC Integrative Epidemiology Unit, University of Bristol, Oakfield House, Oakfield Grove, Bristol BS8 2BN, UK; b School of Social and Community Medicine, University of Bristol, Oakfield House, Oakfield Grove, Bristol BS8 2BN, UK; c Bristol Heart Institute, School of Clinical Sciences, University of Bristol, Bristol, UK

**Keywords:** Mitochondrial DNA, Copy number, ALSPAC, Complex traits, Cardiovascular disease, Diabetes

## Abstract

The mitochondrial genome is present at variable copy number between individuals. Mitochondria are vulnerable to oxidative stress, and their dysfunction may be associated with cardiovascular disease.

The association of mitochondrial DNA copy number with cardiometabolic risk factors (lipids, glycaemic traits, inflammatory markers, anthropometry and blood pressure) was assessed in two independent cohorts of European origin women, one in whom outcomes were measured at mean (SD) age 30 (4.3) years (*N* = 2278) and the second at 69.4 (5.5) years (*N* = 2872). Mitochondrial DNA copy number was assayed by quantitative polymerase chain reaction. Associations were adjusted for smoking, sociodemographic status, laboratory factors and white cell traits.

Out of a total of 12 outcomes assessed in both cohorts, mitochondrial DNA copy number showed little or no association with the majority (point estimates were close to zero and nearly all *p*-values were > 0.01). The strongest evidence was for an inverse association in the older cohort with insulin (standardised beta [95%CI]: − 0.06, [− 0.098, − 0.022], *p* = 0.002), but this association did not replicate in the younger cohort.

Our findings do not provide support for variation in mitochondrial DNA copy number having an important impact on cardio-metabolic risk factors in European origin women.

## Highlights

• MtDNA copy number was studied in relation to cardiometabolic traits in two cohorts of women.
• Associations were adjusted for a number of possible confounders.
• There was weak evidence for an inverse relationship between mtDNA copy number and insulin in the older cohort.
• The findings do not suggest an important association between mtDNA copy number and the majority of phenotypes studied.

## Author summary

Mitochondria are organelles that liberate adenosine triphosphate in order to provide energy for a cell's requirements. Mitochondria contain their own circular genome, which is present at variable copy number between individuals. A number of predominantly small studies have examined the relationship between mitochondrial DNA copy number (mtDNA CN) and cardiometabolic traits. In one of the largest studies of its kind, we have studied mtDNA CN in relation to a variety of cardiometabolic risk factors in two large cohorts of European women. We were able to make use of these rich data resources in order to control for a range of confounding variables, including cellular heterogeneity. We found no consistent evidence to suggest that mtDNA CN was related to the cardiometabolic traits studied, although after considering multiple testing, we did find weak evidence of a positive association with cholesterol in the younger cohort, and an inverse association with insulin in the older cohort. This latter association has been reported more consistently in previous literature. Whilst we cannot rule out associations between mtDNA CN and some cardiometabolic traits, our results do not suggest that variation in mtDNA CN has a major impact on cardio-metabolic risk factors in European origin women.

## 1 Introduction

Mitochondria are organelles responsible for the liberation of energy in the form of adenosine triphosphate (ATP), which is hydrolysed to meet a cell's energy requirements. Mitochondria contain a double-stranded genome of 16.6 kb that encodes 37 genes, including the complexes of the electron transport chain (Dimauro and Davidzon, 2005). The electron transport chain represents the end-point of cellular respiration that facilitates ATP synthesis (Jonckheere et al., 2012).

Each mitochondrion contains a relatively constant (Jonckheere et al., 2012, Ziegler et al., 2015) number of mitochondrial DNA (mtDNA) (Wiesner et al., 1992), yet mitochondria number varies enormously according to cell lineage and function (Robin and Wong, 1988). This translates into considerable interindividual differences in mtDNA content, and overall mtDNA copy number (mtDNA CN) has been observed to decline with age (Short et al., 2005).

Hereditary mutations in nuclear DNA (nDNA) in genes controlling mitochondrial deoxyribonucleoside triphosphate (dNTP) synthesis or replication (reviewed elsewhere (El-Hattab and Scaglia, 2013)) may lead to the autosomal recessive mitochondrial depletion syndromes (MDS). Diseases resulting from mitochondrial depletion may be multisystem, with organs relying heavily on aerobic metabolism (e.g. skeletal/cardiac muscle, liver, brain, kidney) (El-Hattab and Scaglia, 2013) affected most (Chinnery, 2014). Age of onset varies, although symptoms and signs are often most severe in childhood disease (Mattman et al., n.d.).

Mitochondrial pathologies manifest clinically as the consequences of respiratory chain dysfunction (Chinnery, 2014). Mitochondria are the principal producers of reactive oxygen species (ROS), which may damage lipids, proteins and nucleic acids, and mtDNA is exquisitely vulnerable to ROS-induced damage (St John et al., 2007), which may lead to inefficiency of the electron transport chain, and further ROS production (Ziegler et al., 2015).

MtDNA CN has been associated with cardiovascular disease (Moslehi et al., 2012, Chen et al., 2014, Huang et al., 2016) and its risk factors, chronological age (Mengel-From et al., 2014), as well as markers and diseases associated with age, e.g. telomere length (Kim et al., 2012a, Kim et al., 2013, Qiu et al., 2015, Sahin et al., 2011), and frailty (Ashar et al., 2015). Cognitive phenotypes associated with decreased mtDNA CN include cognitive impairment (Lee et al., 2010), dementia (Coskun et al., 2004, Coskun et al., 2012, Gatt et al., 2013, Podlesniy et al., 2013, Rice et al., 2014), and psychiatric morbidities (depression (Kim et al., 2011, Chang et al., 2015) bipolar disorder (Chang et al., 2014, De Sousa et al., 2014), and post-traumatic stress disorder [PTSD]) (Bersani et al., 2016). For detail, see Online Resource 1, Online Resource 2, and Online Resource 3.

Studies have generally found an inverse correlation between mtDNA CN and cardio-metabolic risk factors and outcomes (Kim et al., 2012b, Huang et al., 2011, Antonetti et al., 1995, Chien et al., 2012, Kaaman et al., 2007, Lindinger et al., 2010, Ding et al., 2015), although a number of studies have also found null (Ding et al., 2015, Asmann et al., 2006, Mozhei et al., 2014), tissue-dependent (Hsieh et al., 2011), or results in the opposite direction (Lee et al., 2014, Malik et al., 2009, Weng et al., 2009). Elevated low-density lipoprotein cholesterol (LDLc) has also been associated with decreased mtDNA CN, with the inverse association reported for high-density lipoprotein cholesterol (HDLc) (Lee et al., 2014, Liu et al., 2005), and a recent case-control study found that mtDNA CN was generally lower in coronary heart disease (Chen et al., 2014). However, whilst mtDNA is heritable (Ding et al., 2015), and the majority of studies have conceptualised mtDNA CN as an exposure, reverse causation is possible: recently, an axis proposed between mitochondria, telomeres and p53 has been discussed (Sahin and DePinho, 2012), suggested that telomere dysfunction and attrition may be associated with impaired mitochondrial function (Kim et al., 2013).

The majority of studies relating mtDNA CN to cardiometabolic traits have been of small sample size (in the order of tens to hundreds of patients, with one recent larger study analysing 2077 patients) (Ding et al., 2015). We studied observational associations between mtDNA CN and cardiometabolic risk factors in two large, independent cohorts of European origin: mothers of the Avon Longitudinal Study of Parents and Children (ALSPAC) study, and participants of the British Women's Heart and Health Study (BWHHS) cohort, a population-based cohort of post-menopausal women. Longitudinal associations were studied in ALSPAC, and cross-sectional associations were studied in BWHHS. We used these rich data sources to control for potential confounding variables. Since the relationship between mtDNA CN and leucocyte subtypes is well-documented, and may bias mtDNA CN analyses (Urata et al., 2008, Knez et al., 2016), we also examined the association between mtDNA CN and white cell traits (Pyle et al., 2010).

## 2 Methods

### 2.1 Cohort details

#### 2.1.1 ALSPAC

The Avon Longitudinal Study of Parents and Children is a prospective cohort of mothers and children. Between 1991 and 1992, 14,541 women living in the former county of Avon, UK were recruited during pregnancy, of whom 13,761 were enrolled into the study. Antenatal blood samples were assayed for mtDNA CN in this study. Participants have been followed up longitudinally since recruitment. Outcome data are those measured at the Focus on Mothers Research Clinic, which took place between 2008 and 2011. Further details are available in the cohort profile paper (Fraser et al., 2013), and the study website contains details of available data through a fully searchable data dictionary: http://www.bris.ac.uk/alspac/researchers/data-access/data-dictionary/.

Ethical approval for the study was obtained from the ALSPAC Ethics and Law Committee and the Local National Health Service (NHS) Research Ethics Committees.

#### 2.1.2 BWHHS

The British Women's Heart and Health Study (BWHHS) recruited 4286 women between the ages of 60–79 from UK general practices [https://www.lshtm.ac.uk/eph/ncde/research/bwhhs/]. Blood was collected at baseline interview (1999–2001) after an overnight or minimum 6-h fast. Details of the sampling strategy and available data, including details of follow-up and data linkage, are described elsewhere (Lawlor et al., 2003). Data for the current analysis are those taken or derived at baseline, unless otherwise stated. Ethical approval for the BWHHS was obtained from NHS Research Ethics Committees of individual centres, in addition to the London Medical Research Ethics Committee.

### 2.2 Assay of mitochondrial DNA copy number

ALSPAC mothers' DNA was extracted from whole blood or white cell pellet samples (anticoagulated with EDTA and heparin, respectively) using a phenol-chloroform method. For BWHHS a salting-out procedure was used to extract DNA from K-EDTA whole blood samples (Zabaneh et al., 2011).

MtDNA CN was measured using a quantitative PCR (qPCR) assay that relates the relative copy number of a mitochondrial DNA amplicon [bases 317–381 in the D-loop region] to a nuclear reference gene [*B2M*] (Malik et al., 2011). For details of the assay, see Online Resource 4.

MtDNA CN was calculated as the relative magnitude of the signal from the mitochondrial amplicon to the nuclear amplicon. PCR efficiency values were calculated from standard curves to adjust raw values, and ‘calibrator’ DNAs were amplified on each microplate, in order to generate a ‘calibration factor’ for each batch. This factor was applied to the previously calculated copy numbers, resulting in efficiency- and calibrator- adjusted value for mtDNA CNs.

### 2.3 Outcome variables

Unless otherwise stated, outcomes were measured at baseline in BWHHS, and at the Focus on Mothers Research Clinic (2008–2011) in ALSPAC.

#### 2.3.1 Anthropometric variables

After measuring height and weight, BMI was calculated as weight (kg)/height (m^2^). Averages of two measures of waist and hip circumference were calculated, and waist-to-hip ratio was derived.

#### 2.3.2 Blood pressure

Systolic and diastolic blood pressure (BP) was measured in both ALSPAC and BWHHS.

#### 2.3.3 Biomarkers

Fasting lipids (total cholesterol, HDLc, LDLc, triglycerides, fasting glucose (all mmol/L), insulin (u/mL) and C-reactive protein (CRP) (mg/L) were measured in ALSPAC and BWHHS. Data on interleukin-6 (IL6) (pg/mL) were also available for BWHHS. Descriptive summaries are given in Table 1.

**Table 1:** Characteristics of BWHHS and ALSPAC cohorts.

Variable	ALSPAC				BWHHS				
	Units	Mean/Median^a^	SD/IQR^a^	N	Units	Mean/Median^a^	SD/IQR^a^	N	P^e^
mtDNA	ratio	45.5^d^	37.9–56.4^d^	2278	ratio	25.8	20.4–31.7	2872	< 1e-06
DNA concn. b	ng/uL	158	76–304	2278	ng/uL	300	210–407	2872	< 1e-06
DNA source	% whole blood samples	39.6		2278					
Age (baseline)	years	30	4.3	2278	years	69.4	5.5	2872	< 1e-06
Education	%≤ GCSE	51.4		2278	score	− 0.0643	0.89	2872	
Occupation	% manual	14		2278	% manual	54.9		2872	< 1e-06
Smoking	% current	20.5		2278	% current	9.64		2872	< 1e-06
Total cholesterol^b^	mmol/L	4.83	4.32–5.4	2050	mmol/L	6.6	5.9–7.4	2854	< 1e-06
HDL cholesterol^b^	mmol/L	1.44	1.2–1.7	2050	mmol/L	1.6	1.4–2	2850	< 1e-06
LDL cholesterol^b^	mmol/L	2.9	2.41–3.43	2050	mmol/L	4.1	3.4–4.8	2793	< 1e-06
Triglycerides^b^	mmol/L	0.88	0.68-1.17	2050	mmol/L	1.6	1.2–2.22	2854	< 1e-06
Fasting glucose^b^	mmol/L	5.15	4.91–5.44	2050	mmol/L	5.8	5.4–6.2	2847	< 1e-06
Insulin^b^	u/mL	4.57	3.23-6.75	2044	u/mL	6.4	4.4–9.9	2859	< 1e-06
Interleukin-6^b^					pg/mL	2.12	1.47–3.11	2796	
C-reactive protein^b^	mg/L	0.98	0.48–2.1	2050	mg/L	1.92	0.948–4.13	2680	< 1e-06
Body Mass Index	kg/m^2	25.4	22.8–29	2266	kg/m^2^	26.7	24–30.1	2841	< 1e-06
Waist-Hip Ratio	ratio	0.809	0.065	2267	ratio	0.819	0.068	2827	< 1e-06
Height	cm	164	6.1	2269	cm	159	6.1	2842	< 1e-06
Systolic BP	mmHg	118	12	2214	mmHg	147	25	2860	< 1e-06
Diastolic BP	mmHg	71.4	7.9	2214	mmHg	79.4	12	2860	< 1e-06
Lymphocytes^b^	Lymphocyte proportion	0.261	0.091	491	All × 10^3^/ml	1.97	1.57–2.43	2872	
Granulocytes^c^	All proportion	0.683	0.083	491	Neut. × 10^3^/mL	4.14	3.36–5.14	2872	
					Baso. × 10^3^/mL	0.06	0.04–0.08	2872	
					Eos. × 10^3^/mL	0.13	0.08–0.19	2872	
Monocytes^c^	All proportion	0.0782	0.022	491	× 10^3^/mL	0.3	0.2–0.4	2872	
Platelets					× 10^3^/mL	281	71	2872	

Other abbreviations: mtDNA = *Z*-scored, log-transformed mtDNA copy number; conc. = concentration; HDL, LDL = High-, low-density lipoprotein; BP = blood pressure; GCSE = General Certificate of Secondary Education (or ‘O’-level); Neut. = Neutrophils; Baso. = Basophils; Eos. = Eosinophils.

a Means (standard deviations [SD]) are shown for continuous variables, medians (interquartile range [IQR]), if skewed.

b Variable skewed.

c Skewed in BWHHS only.

d Calculated using efficiency-adjusted crossing points (C_p_s) for the nuclear and mitochondrial amplicons, using the equation: mtDNA CN = (2^(nuclear DNA Cp – mitochondrial DNA Cp)).

e P values are for either Mann-Whitney *U* (Skewed continuous variables), independent *t*-tests (for continuous normal variables, assuming unequal variance for age), or chi-square test (categorical variables).

### 2.4 Confounding variables

#### 2.4.1 Sociodemographic variables

Age, as well as smoking and socioeconomic factors are associated with cardiovascular disease (Lawlor et al., 2005). mtDNA has been observed to decline with age (Mengel-From et al., 2014) and may also be associated with smoking and socioeconomic position (Hosnijeh et al., 2014), and for these reason, these variables were chosen as possible confounders.

For ALSPAC, age was recorded as age at delivery, which approximated age at DNA sampling. Age at baseline was available for BWHHS. Analyses were restricted to women of self-reported ‘white’ ethnicity.

In ALSPAC, smoking was quantified as the number of cigarettes smoked per day shortly pre-pregnancy, (0, 1–4, 5–9, 10–14, 15–19, 20–24, 25–29, > 30). Smoking was quantified at baseline in BWHHS (0 [never smoker], 0 [ex-smoker], 1–9, 10–19, 20–29, > 30). For descriptive analyses, smoking is recoded into smokers/‘non-smokers.

In ALSPAC, highest education level was divided into: Vocational, CSE (Certificate of Secondary Education, ‘O’ (Ordinary) Level, ‘A’ (Advanced) level, or degree. Four categories of the Standard Occupational Classification classified occupational class: I, II, III [non-manual], plus a collapsed category (III [manual], IV, V, and Armed Forces) (Office for National Statistics, E. L, et al., 2000). For BWHHS, continuous measures of employment and education score by neighbourhood were used (according to the Indices of Deprivation [2000]), where a higher score indicates greater deprivation, except in descriptive tables, wherein occupation was classified as for ALSPAC (with those missing occupational status data coded to ‘manual’) (Indices of Deprivation 2000, 2000).

#### 2.4.2 DNA concentration and source

DNA sample concentration (ng/μl) was measured by a PicoGreen® fluorescence-based method. We have observed a negative correlation between the obtained mtDNA CN and initial stock DNA concentration, from which working concentrations were prepared (Malik et al., 2011), and so controlled for this in our analyses. Since we observed a lower mtDNA CN in whole blood samples, which may be due to differing cell proportions, we also adjusted for this covariate, as DNA was from either whole blood or white cell pellets in ALSPAC.

#### 2.4.3 Cell counts

Different white cell lineages may contain different numbers of mitochondria, and hence mtDNA: (Pyle et al., 2010) e.g., neutrophils contain a relative paucity of mitochondria compared to lymphocytes. Platelets, which are anucleate, and yet mitochondria-rich, may also alter the interpretation of assayed mtDNA CNs (Urata et al., 2008). In order to assess the relationship between mtDNA CN and cardiometabolic risk factors independent of cellular heterogeneity, latter models adjusted for cell populations. Cell counts were not directly assayed in ALSPAC. However, the ‘ARIES’ subset (Relton et al., 2015) of the cohort have been assayed using the Illumina Infinium HumanMethylation450 BeadChip (Illumina 450 K) array. DNA methylation profiles may be used in order to infer proportions of various cellular populations in samples assayed on high dimension methylation arrays, such as the Illumina 450 K array. The most popular algorithm to achieve this goal is the ‘Houseman’ method (Houseman et al., 2012), which uses individual ‘methylation signatures’ of samples as a proxy for the distribution of white cells in combination with a training set of samples of purified white cells. This method has been applied previously to individuals belonging to the ARIES subset of ALSPAC (Richmond et al., 2015), implemented using the ‘estimateCellCounts’ function of the R package ‘minfi’ (Jaffe and Irizarry, 2014). Proportions of CD4 + and CD8 + T lymphocytes (CD4T, CD8T), B lymphocytes, Natural Killer (NK) cells, Monocytes, and Granulocytes were estimated. One variable for lymphocyte proportion was derived by adding CD8T, CD4T, and B- lymphocyte proportions (NK cells were not included so as to avoid perfect prediction) and we used this to adjust for cell type.

In BWHHS, absolute count of platelets, basophils, eosinophils, neutrophils, lymphocytes and monocytes were assayed at baseline.

### 2.5 Missing data

MtDNA CN data from 8158 ALSPAC and 3673 BWHHS women were available. 2278 ALSPAC and 2872 BWHHS participants also had data on age at DNA sampling, DNA concentration, occupational class, education level, smoking, plus ≥ 1 outcome variable. In addition, all 2872 BWHHS women had cell count data. Cell proportion data were only available for a subset of the 2278 ALSPAC women [max *N* = 491].

Four ALSPAC and four BWHHS participants were removed from the analysis as they had severely outlying values, either considered implausible and/or as biasing regression estimates by excessive leverage.

### 2.6 Statistical analysis

Table 1 shows descriptive data. Means (standard deviations, SD) are presented for normally distributed data. In the case of non-normality, medians (interquartile ranges, IQR) are shown. Means are compared with independent sample *t*-tests (assuming unequal variance for ‘age’, given the uniform sampling in BWHHS); medians are compared with the Mann-Whitney *U* test. Categorical variables were compared using a chi-squared test (1 degree of freedom). Haematological variables were not comparable, as ALSPAC data were estimated cell proportions, whereas BWHHS data were cell counts.

To assess relationships between mtDNA CN and covariates in each cohort, independent sample t-tests were performed for binary covariates, and Pearson's correlation coefficients were computed for continuous (log-transformed variables were used in cases of positive skew) (see Table 2, Table 3).

**Table 2:** Comparison of mean log-transformed, z-scored mtDNA (SD) between possible confounding variables in ALSPAC and BWHHS.

Confounder	ALSPAC							BWHHS						
	Effect size measure	Risk group	Effect size	95%LCI	95%UCI	P	N	Effect size measure	Risk group	Effect size	95%LCI	95%UCI	P	N
DNA concn.^a^	Pearson correlation		− 0.170	− 0.209	− 0.130	3.28e-16	2278	Pearson correlation		− 0.229	− 0.263	− 0.194	< 2e-16	2872
DNA source	Mean difference	Whole blood vs. WC pellet	− 0.367	− 0.447	− 0.287	< 2e-16	2278							
Age at sampling	Pearson correlation		0.033	− 0.009	0.074	0.121	2278	Pearson correlation		− 0.018	− 0.054	0.019	0.346	2872
Education	Mean difference	≤ GCSE vs. > GCSE	0.016	− 0.064	0.095	0.701	2278	Pearson correlation		− 0.097	− 0.133	− 0.061	1.77e-07	2872
Occupation	Mean difference	Manual vs. non-manual	− 0.008	− 0.123	0.106	0.886	2278	Mean difference	Manual vs. non-manual	− 0.102	− 0.175	− 0.029	0.006	2872
Smoking	Mean difference	Smoker vs. non-smoker	− 0.072	− 0.170	0.027	0.153	2278	Mean difference	Smoker vs. non-smoker	− 0.060	− 0.184	0.064	0.341	2872

Abbreviations: concn. = Concentration; WC = white cell; GCSE = General Certificate of Secondary Education; 95% LCI/UCI = Lower and upper 95% confidence interval bounds.

a log-transformed.

**Table 3:** Pearson's correlation coefficients between haematological parameters and log z-scored mtDNA CN in ALSPAC and BWHHS.

Cell type	ALSPAC^a^							BWHHS						
	Subtype	Units	r	95%LCI	95%UCI	P	N	Subtype	Units	r	95%LCI	95%UCI	P	N
Lymphocytes^b^	All	Proportion	0.279	0.195	0.359	3.18e-10	491	All	× 10^3^/ml	0.159	0.123	0.195	9.90e-18	2872
Granulocytes^c^	All	Proportion	− 0.362	− 0.436	− 0.282	1.22e-16	491	Neutrophils**	× 10^3^/mL	− 0.072	− 0.108	− 0.035	1.14e-04	2872
								Basophils**	× 10^3^/mL	0.055	0.018	0.091	3.24e-03	2872
								Eosinophils**	× 10^3^/mL	0.047	0.010	0.083	1.25e-02	2872
Monocytes^c^		Proportion	0.125	0.037	0.212	5.39e-03	491		× 10^3^/mL	0.087	0.051	0.123	2.98e-06	2872
Platelets^c^									× 10^3^/mL	0.062	0.026	0.098	8.77e-04	2872

Abbreviations: r = Correlation coefficient, 95% LCI/UCI = Lower and upper 95% confidence interval bounds.

a Cell count data are estimated from Illumina 450 k Methylation data for ALSPAC, and for BWHHS, data are from actual full blood count assays. In ALSPAC, DNA is extracted from either white cells or whole blood, whereas all BWHHS DNA is extracted from whole blood.

b log-transformed.

c log-transformed in BWHHS only.

For the main analyses, positively skewed outcomes were log-transformed where this led to a better approximation of normality. Outcomes were then z-scored to yield standardised regression coefficients. Waist-hip ratio, height, and BP were not transformed as their distributions were approximately normal.

Regression analyses of *Z*-scored outcomes on Z log mtDNA are presented for ALSPAC and BWHHS. M1 was unadjusted, and M2 was adjusted for age at DNA sampling (continuous), education (categorical for ALSPAC, continuous for BWHHS), smoking status (assuming linearity over categories), occupational class (categorical), DNA concentration (continuous) and DNA source (categorical, ALSPAC only). M3 additionally adjusted for cell counts (as estimated proportions [ALSPAC], and counts [BWHHS] — see Table 3).

With the exception of mtDNA CN (which was logged to facilitate Z-scoring, with z-scores for mtDNA CN being calculated before merging with phenotype data), none of the other independent variables in Table 2, Table 3 were log-transformed for the purpose of regression models. Residual-versus-fitted values plots and quantile-quantile plots were examined, in order to check assumptions of normally distributed residuals, homoscedasticity, and linearity.

Sensitivity analyses were carried out for ALSPAC, which fitted models M1-M3 separately for those ALSPAC Mothers with DNA extracted from white cells and whole blood.

Both fixed- and random-effects meta-analyses of the ALSPAC and BWHHS results were performed using the R package ‘meta’.

Analyses were undertaken using R 3.2.4.

### 2.7 Multiple testing

Nyholt's method of spectral decomposition was used to calculate the effective number of independent tests from correlation matrices of outcomes (calculated separately for ALSPAC and BWHHS) (Nyholt, 2004). The *p* values presented are not corrected for multiple testing, but the number of independent tests are reported for reference.

## 3 Results

### 3.1 Descriptives

Descriptive statistics for ALSPAC and BWHHS are shown in Table 1. The mean (SD) age of participants was 69.4 (5.5) years in BWHHS, and 30.0 (4.3) years in ALSPAC. ALSPAC women were more likely to smoke (yet 32.7% of BWHHS women were ex-smokers). Fewer in ALSPAC had a manual occupation, and ALSPAC mothers were educated to a higher level. ALSPAC participants had healthier levels of cardiovascular risk factors (including lower total cholesterol, LDLc, fasting glucose and insulin); they were also taller and leaner, with lower blood pressure.

Median (IQR) mtDNA CN was higher in ALSPAC women: 45.5 (37.9–56.4) compared to 25.8 (20.4–31.7) in BWHHS (Mann Whitney *U p* < 1e-06). However, DNA extraction method differed between the two cohorts (phenol for ALSPAC, salting-out for BWHHS) and so these differences may partially be attributable to laboratory factors.

### 3.2 Confounding variables and laboratory covariates

Relationships between potential confounding variables, laboratory covariates and mtDNA CN (all subsequent analyses use *Z*-scores of logged mtDNA) are shown in Table 2. DNA concentration was inversely associated with mtDNA in both cohorts (mean difference: − 0.170 [− 0.209, − 0.130], *p* = 3.28e-16 [ALSPAC]; − 0.229 [− 0.263, − 0.194], *p* ≤ 2e-16 [BWHHS]). In ALSPAC, mtDNA CN was lower in those women from whom DNA was extracted from whole blood (mean difference: − 0.367 [− 0.447, − 0.287], *p* < 2e-16).

Whilst mtDNA CN was lower in BWHHS (see Table 1, *p* < 1e-06 for independent *t*-test assuming unequal variance), there was no evidence of an association between mtDNA count and age within ALSPAC or BWHHS, nor with and smoking. There was a negative association between socioeconomic status and mtDNA CN in BWHHS (Pearson's R: − 0.097 [95%CI: − 0.133, − 0.061], *p* = 1.77e-07 for education score, where a higher score indicates lower level of education); mean difference: -0.102 [95%CI:-0.175, − 0.029], *p* = 0.006 for manual occupation), but not in ALSPAC.

The correlations of mtDNA CN with haematological parameters varied by cell type (see Table 3): positive associations were observed for proportions (in ALSPAC) and counts (in BWHHS) of lymphocytes and monocytes (although monocytes had considerably stronger evidence of association in BWHHS), and for additional counts assayed only in BWHHS (platelet, basophil and eosinophil count). Negative associations were observed between mtDNA and granulocyte proportion in ALSPAC, and neutrophil count in BWHHS (notably, neutrophils make up the majority of circulating granulocytes). Although cell data were proportions for ALSPAC and counts for BWHHS, effect sizes were similar in both cohorts (see Table 3).

### 3.3 Regression models

The spectral decomposition method of Nyholt (2004) estimated that 10 and 11 independent tests were carried out in ALSPAC and BWHHS, respectively.

Out of a total of 12 outcomes assessed in both cohorts, mtDNA showed little or no association with the majority (point estimates were close to zero, or attenuated after adjustment).

#### 3.3.1 ALSPAC

Before adjustment for cell counts, there was weak evidence of a positive association with waist-hip ratio and mtDNA CN (standardised beta: [95% CI]: 0.060 [0.017, 0.104], *p* = 0.007). After adjusting for cell counts (M3) there was very weak evidence of a positive association between mtDNA CN and total cholesterol (standardised beta: [95% CI]: 0.098 [− 0.008, 0.204], *p* = 0.070) (see Table 4). However, this association was also consistent with the null effect, and it did not replicate in BWHHS.

**Table 4:** Standardised linear regression of cardiovascular traits on mtDNA CN (ALSPAC).

Outcome	M1					M2					M3				
	B	LCI	UCI	P	N	B	LCI	UCI	P	N	B	LCI	UCI	P	N
Cholesterol^a^	0.061	0.016	0.106	0.008	2050	0.058	0.012	0.103	0.013	2050	0.098	− 0.008	0.204	0.070	465
HDL^a^	0.022	− 0.024	0.067	0.348	2050	0.015	− 0.031	0.061	0.535	2050	− 0.005	− 0.109	0.099	0.929	465
LDL^a^	0.036	− 0.009	0.081	0.118	2050	0.037	− 0.009	0.083	0.117	2050	0.069	− 0.038	0.177	0.206	465
Triglycerides^a^	0.055	0.010	0.101	0.016	2050	0.046	− 0.001	0.092	0.054	2050	0.083	− 0.025	0.192	0.133	465
Glucose^a^	0.019	− 0.026	0.065	0.401	2050	0.022	− 0.025	0.069	0.350	2050	0.049	− 0.056	0.154	0.362	465
Insulin^a^	0.012	− 0.033	0.057	0.603	2044	0.022	− 0.025	0.069	0.360	2044	0.014	− 0.094	0.122	0.796	464
C-reactive protein^a^	0.015	− 0.030	0.060	0.513	2050	0.012	− 0.035	0.058	0.615	2050	− 0.054	− 0.165	0.056	0.336	465
Body Mass Index^a^	− 0.010	− 0.052	0.033	0.656	2266	− 0.004	− 0.048	0.040	0.862	2266	− 0.098	− 0.205	0.009	0.074	488
Waist-Hip Ratio	0.052	0.009	0.094	0.018	2267	0.060	0.017	0.104	0.007	2267	− 0.005	− 0.108	0.099	0.929	489
Height	− 0.021	− 0.064	0.021	0.330	2269	− 0.021	− 0.065	0.023	0.350	2269	− 0.046	− 0.151	0.060	0.397	489
Systolic BP	0.025	− 0.018	0.068	0.260	2214	0.027	− 0.018	0.071	0.241	2214	− 0.048	− 0.153	0.056	0.363	473
Diastolic BP	0.010	− 0.033	0.053	0.650	2214	0.015	− 0.030	0.060	0.512	2214	− 0.069	− 0.173	0.035	0.196	473

Abbreviations: M1 = Model 1 (unadjusted); M2 = Model 2 (adjusted for age at DNA sampling, DNA source, education level, smoking status, occupational class, DNA concentration); M3 = Model 3 (as M2, plus adjustment for cell counts as described in Table 3). B = Standardised beta coefficient; LCI = 95% confidence interval (lower bound); UCI = 95% confidence interval (upper bound); HDL, LDL = High-, Low-density lipoprotein cholesterol. BP = blood pressure.

a log-transformed.

Sensitivity analyses for ALSPAC repeated models M1–M3, but were stratified by whether women had DNA extracted from white cells, or whole blood (see Online Resource 5 for results). After stratification, the results were broadly consistent with the combined analyses in Table 4. After restricting the analysis to whole blood samples, mtDNA CN was weakly associated with total cholesterol, LDLc and triglycerides. Although this association did not survive cell-proportion adjustment, this is likely to be a function of power. Whilst these effect sizes were directionally concordant in the (larger) white cell analysis, they were also consistent with the null.

#### 3.3.2 BWHHS

In BWHHS, the most consistent relationship that survived adjustment in the final model, M3, was a negative association between mtDNA and insulin (standardised beta [95%CI]: − 0.060 [− 0.098, − 0.022], *p* = 0.002) (see Table 5), but again, there was no evidence of this association in ALSPAC.

**Table 5:** Standardised linear regression of cardiovascular traits on mtDNA CN (BWHHS).

Outcome	M1					M2					M3				
	B	LCI	UCI	P	N	B	LCI	UCI	P	N	B	LCI	UCI	P	N
Cholesterol^a^	0.031	− 0.006	0.067	0.102	2854	0.031	− 0.006	0.069	0.102	2854	0.013	− 0.025	0.051	0.510	2854
HDL^a^	− 0.055	− 0.092	− 0.018	0.003	2850	− 0.058	− 0.095	− 0.021	0.002	2850	− 0.020	− 0.058	0.017	0.284	2850
LDL^a^	0.055	0.018	0.092	0.004	2793	0.041	0.003	0.079	0.033	2793	0.024	− 0.014	0.063	0.214	2793
Triglycerides^a^	− 0.005	− 0.042	0.031	0.774	2854	0.028	− 0.009	0.065	0.136	2854	− 0.019	− 0.056	0.018	0.316	2854
Glucose^a^	− 0.028	− 0.065	0.009	0.132	2847	− 0.019	− 0.056	0.019	0.330	2847	− 0.023	− 0.062	0.015	0.234	2847
Insulin^a^	− 0.058	− 0.095	− 0.021	0.002	2859	− 0.034	− 0.072	0.003	0.073	2859	− 0.060	− 0.098	− 0.022	0.002	2859
Interleukin-6^a^	− 0.022	− 0.059	0.015	0.240	2796	0.013	− 0.024	0.050	0.484	2796	− 0.001	− 0.037	0.034	0.938	2796
C-reactive protein^a^	− 0.024	− 0.062	0.014	0.212	2680	0.003	− 0.036	0.042	0.876	2680	− 0.006	− 0.043	0.032	0.771	2680
Body Mass Index^a^	0.027	− 0.010	0.064	0.147	2841	0.046	0.008	0.083	0.017	2841	0.016	− 0.022	0.054	0.421	2841
Waist-Hip Ratio	− 0.016	− 0.053	0.021	0.386	2827	0.004	− 0.034	0.041	0.854	2827	− 0.021	− 0.060	0.017	0.271	2827
Height	− 0.011	− 0.048	0.025	0.546	2842	− 0.032	− 0.069	0.004	0.085	2842	− 0.028	− 0.065	0.009	0.144	2842
Systolic BP	− 0.036	− 0.073	0.000	0.051	2860	− 0.019	− 0.055	0.017	0.297	2860	− 0.033	− 0.070	0.004	0.079	2860
Diastolic BP	− 0.042	− 0.078	− 0.005	0.026	2860	− 0.033	− 0.070	0.004	0.082	2860	− 0.041	− 0.079	− 0.003	0.036	2860

Abbreviations: M1 = Model 1 (unadjusted); M2 = Model 2 (adjusted for age at DNA sampling, education level, smoking status, occupational class, DNA concentration); M3 = Model 3 (as M2, plus adjustment for cell counts as described in Table 3). B = Standardised beta coefficient; LCI = 95% confidence interval (lower bound); UCI = 95% confidence interval (upper bound); HDL, LDL = High-, Low-density lipoprotein cholesterol; BP = blood pressure.

a log-transformed.

#### 3.3.3 Meta-analysis

**Fig. 1:**
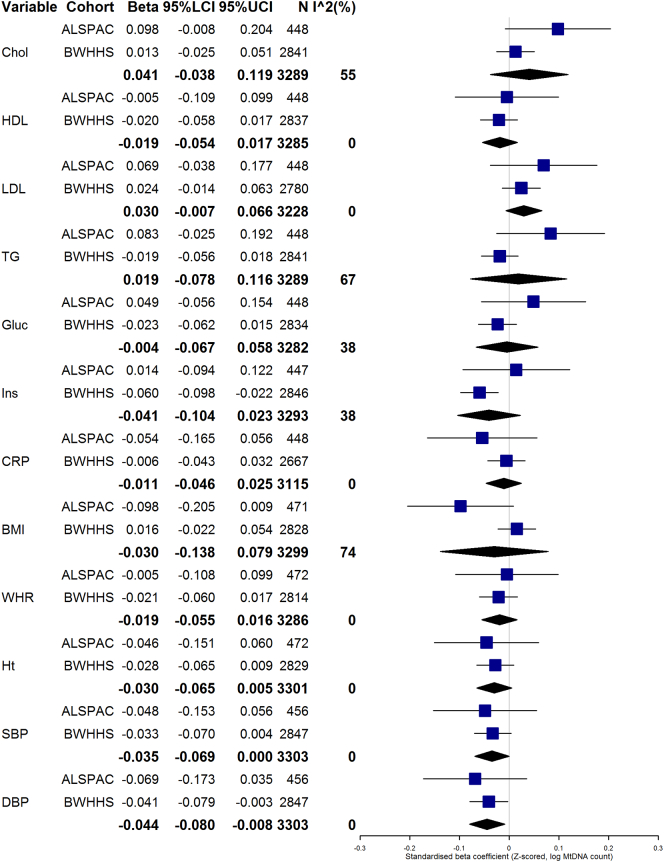
Random-effects meta-analysis of model M3. Forest plot showing random-effects meta-analyses of fully-adjusted model (M3) of associations observed in ALSPAC and BWHHS. For reference, the effect sizes of the individual associations between mtDNA and outcome variables in ALSPAC and BWHHS are also shown on the forest plots, with meta-analysis summary estimates shown as diamonds. Abbreviations: Chol = total cholesterol; HDL = High-density LDL cholesterol; LDL = Low-density lipoprotein cholesterol; TG = triglycerides; Gluc = Fasting glucose; Ins = Insulin; CRP = C-reactive protein; BMI = Body Mass Index; WHR = Waist-Hip Ratio; Ht = height; SBP = systolic blood pressure; DBP = Diastolic blood pressure; 95%LCI/UCI = lower and upper bounds of 95% confidence interval. N = Sample size, I^2 = I^2^ statistic for heterogeneity.

**Fig. 2:**
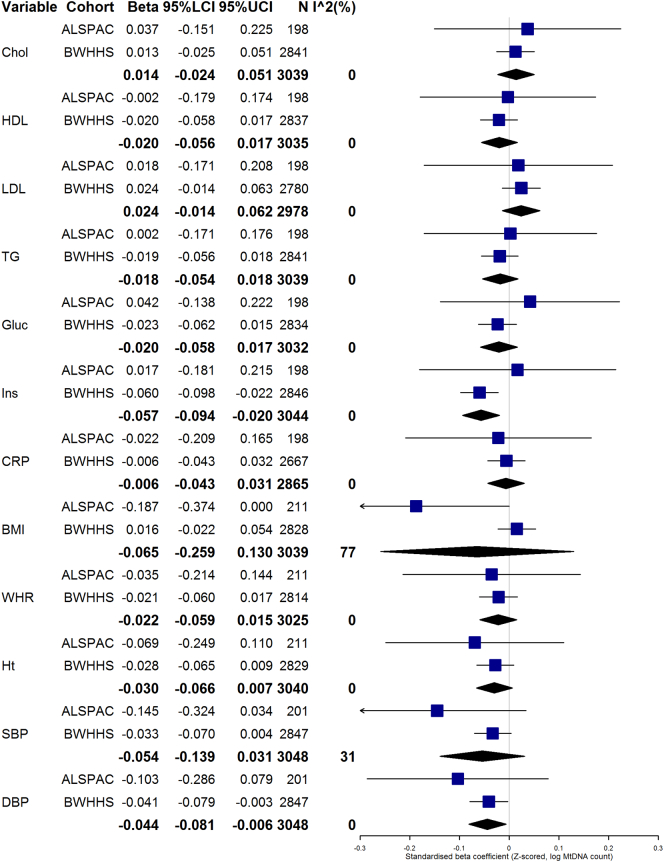
Random-effects meta-analysis of model M3 (sensitivity). Forest plot showing random-effects meta-analyses of fully-adjusted model (M3) of associations observed in ALSPAC (whole blood samples only) and BWHHS. For reference, the effect sizes of the individual associations between mtDNA and outcome variables in ALSPAC and BWHHS are also shown on the forest plots, with meta-analysis summary estimates shown as diamonds. Abbreviations: Chol = total cholesterol; HDL = High-density LDL cholesterol; LDL = Low-density lipoprotein cholesterol; TG = triglycerides; Gluc = Fasting glucose; Ins = Insulin; CRP = C-reactive protein; BMI = Body Mass Index; WHR = Waist-Hip Ratio; Ht = height; SBP = systolic blood pressure; DBP = Diastolic blood pressure. 95%LCI/UCI = lower and upper bounds of 95% confidence interval. N = Sample size, I^2 = I^2^ statistic for heterogeneity.

Results of random-effects analyses for ALSPAC Mothers and BWHHS are shown in Fig. 1, Fig. 2(and additionally tabulated in Online Resource 6). Fig. 2 only combines those with mtDNA CN assayed from whole blood (all of BWHHS and a subset of ALSPAC Mothers). After meta-analysing all participants in model 3, there was weak evidence of a negative association between systolic and diastolic blood pressure and Z log mtDNA in fixed-effects analyses (I^2^ = 0%) (see Fig. 1). On restricting the analysis to just those ALSPAC Mothers with mtDNA CN assayed from whole blood, heterogeneity was generally reduced, but associations were broadly similar. In this analysis (see Fig. 2), there was still some evidence for an association of mtDNA CN with diastolic blood pressure, and additionally, more evidence for a negative association with insulin (beta: [95% CI]: − 0.057 [− 0.094, 0.020], *p* = 0.003, I^2^ = 0). However, it should be noted that an even smaller number of ALSPAC women contributed to this analysis, so the associations were primarily driven by BWHHS.

## 4 Discussion

This is one of the largest studies to examine the association of mitochondrial copy number (as measured by a quantitative qPCR assay) with a range of cardiometabolic traits in general population samples. In addition, it is amongst a relatively small number of studies to have controlled for haematological parameters (Knez et al., 2016, Tin et al., 2016).

We observed little evidence for associations of cardiometabolic traits with mtDNA CN. A negative association between mtDNA CN and insulin was unique to BWHHS. The trend observed for the association with lipids in ALSPAC (a positive relationship between mtDNA and total cholesterol) is at odds with the direction of association reported in the literature (Huang et al., 2011). There was also a trend towards an inverse association between mtDNA CN and BMI (observed in ALSPAC only). The association between high insulin and low mtDNA is concordant with previous observations that diabetes and insulin resistance are generally associated with lower mtDNA CN (Chien et al., 2012, Hsieh et al., 2011, Rolo and Palmeira, 2006). Although we did not observe this same association in the ALSPAC mothers, the cohorts are of different ages; it is therefore possible that a lower prevalence of insulin resistance amongst the younger population is the reason that we did not observe the association in these individuals. We additionally saw weak evidence of an association between higher mtDNA CN and lower blood pressure after meta-analysis, but this did not survive multiple testing correction. Overall, the small number of associations that we see do not replicate within our cohorts or with published literature and suggest that beyond chance there is little evidence that mtDNA CN is an important determinant or predictor of cardio-metabolic risk in European origin women. Despite this, some studies have observed that mtDNA is related to sex (Ashar et al., 2015, Purdue et al., 2012), and ethnicity (Purdue et al., 2012). Whilst our cohorts are comparable to one another in terms of these characteristics, it is possible that our results may not necessarily be generalisable to other populations, for example, if there are interactions between mtDNA CN and cardiometabolic risk factors according to these characteristics.

The literature studying mtDNA and components of the metabolic syndrome has been reported in such a way that suggests inconsistency: in those papers studying metabolic syndrome, hyperlipidaemia (of LDLc) or adiposity, most reported an inverse association (Kim et al., 2012b, Huang et al., 2011, Kaaman et al., 2007, Lindinger et al., 2010, Mozhei et al., 2014, Lee et al., 2014), with some reporting the opposing direction (Lindinger et al., 2010, Lee et al., 2014). Results are more equivocal in the diabetes literature, with positive (Malik et al., 2009, Weng et al., 2009), inverse (Antonetti et al., 1995, Chien et al., 2012, Hsieh et al., 2011) and no associations (Lindinger et al., 2010) reported. Notably, the literature contains cross-sectional, longitudinal, and tissue-specific studies, and both clinical (such as patients with diabetes) and general population samples (e.g. studying hyperglycaemia in a population sample); it is plausible that the results from clinical samples are not generalisable to the associations observed at the population level. Finally, the sample sizes of the majority of papers are small, it is difficult to discern whether the variation reported in the literature represents true heterogeneity of effect, or chance variation around a null effect.

Since mitochondrial load varies by leucocyte subtype, we assessed the relationship between mtDNA count and blood cell data. Platelets contain no nDNA, yet an abundance of mitochondria, and thus may artificially inflate mtDNA CN, as reported previously (Urata et al., 2008). Neutrophils reportedly contain up to 10–15 times fewer mitochondria than peripheral blood mononuclear cells (PBMCs) (Maianski et al., 2004), and accordingly, they exhibited a negative relationship with mtDNA count. PBMCs are mitochondria rich, with ‘granulocyte mtDNA CN observed as lower than lymphocyte and monocyte copy number’ (Pyle et al., 2010). Importantly, despite having absolute counts in BWHHS but only estimated proportions in ALSPAC, the direction and magnitude of the associations between cell types and mtDNA CN were comparable.

Whilst these results demonstrate how important it may be to control for cell populations when performing mtDNA association analyses, cell counts could also mediate an association between mtDNA and cardiovascular disease, via inflammation (white cells, in particular macrophages, have a causal role in atherosclerosis) (Yu et al., 2013). In this case, controlling for these variables may be overly conservative.

There are several limitations to this work. Whilst the mtDNA assay was undertaken by one individual using identical protocols in both studies, DNA extraction was undertaken with different methods; by a phenol-chloroform method in ALSPAC and by ‘salting out’ in BWHHS. We computed *Z*-scores so that the magnitude of effect sizes between the two cohorts were comparable, yet nonetheless, if extraction method has an effect on the precision or linearity of the mtDNA assay, this could introduce bias, though the consistent null associations for the vast majority of results between the two studies suggests major bias from this source is unlikely. Furthermore, mtDNA CN was assayed from either whole blood or white cell DNA samples in ALSPAC. Whilst we controlled for sample type as a covariate in models M2 and M3, it is possible that this could bias our results, since we were not able to control for other cell populations in the ALSPAC whole blood sample, such as platelets. Nevertheless, we did not observe major differences between the directions of associations observed in a sensitivity analysis that stratified the ALSPAC sample by whether mtDNA CN was assayed from whole blood or white cells, although the heterogeneity (quantified by the I^2^-statistic) (Higgins et al., 2003) in our meta-analytic estimates did decrease for some variables in this analysis, and the weak lipid association in ALSPAC was primarily driven by the whole blood samples. In addition, the sample size for ALSPAC was comparatively small, and the cell proportion measures only estimated, albeit by a previously validated method with an accuracy ‘within 10%, and often less than 5%.’ (Houseman et al., 2012) To minimise measurement error in the mtDNA CN, a calibrator was used to control for between-plate effects. Moreover, we controlled for a wide array of variables (laboratory covariates, sociodemographic confounders, and haematological parameters) which may have explained the observed associations. Nonetheless, it is possible that the association with total cholesterol in ALSPAC may be due to residual confounding, for example, due to measurement error in the estimated cell proportions.

It is important to note that we studied peripheral blood mtDNA CN, and cannot necessarily extrapolate our findings to those which we might have observed, had we studied target tissues of interest (e.g. cardiomyocytes, endothelial cells). In animal studies, some correlation has been found between mtDNA CN assayed in the blood, adipose tissue, liver and mammary gland of dairy cows, suggesting that in this case, blood mtDNA CN is an appropriate proxy for other tissues (Laubenthal et al., 2016). However, clearly these results may not necessarily apply to human subjects. Nevertheless, human studies have successfully used peripherally assayed mtDNA CN to proxy for a tissue of interest; Pyle et al. found concordant depletion of mtDNA CN in peripheral white blood cells, as well as substantia nigra tissue from patients with Parkinson's disease (Pyle et al., 2016). Moreover, Huang et al. observed depletion of mtDNA CN in a case-control study of cardiac failure, and noted a strong (Pearson's *r* = 0.718) correlation between mtDNA CN assayed in blood and in cardiomyocytes, although their sample was small (Huang et al., 2016). From these studies, we might therefore speculate that our results should be applicable to tissues of relevance to cardiovascular disease, although we cannot state this with certainty.

Whilst this paper only studied mtDNA CN, an extension of our work could be to examine associations between mtDNA sequence variation (e.g. single-nucleotide polymorphisms, [SNPs], and structural variants) in relation to cardiometabolic risk factors. MtDNA haplogroups have also been analysed in relation to coronary artery disease (Kofler et al., 2009, Chinnery et al., 2010), diabetes (Chinnery et al., 2007), lipid profiles (Hulgan et al., 2011), blood pressure (Rea et al., 2013), obesity (Nardelli et al., 2013), CRP (Kofler et al., 2009), and many others (Samuels et al., 2006), but consensus amongst findings is limited, and well-powered studies are needed to detect these effects (Samuels et al., 2006). Accordingly, very large studies have had success in replicating certain associations between mtDNA SNPs (and haplogroups) and age-related disease (Hudson et al., 2014). The 4977 bp deletion (Cortopassi and Arnheim, 1990) has been related to cardiac endpoints (Corral-Debrinski et al., 1992) (although conclusions vary (Arai et al., 2003)), but whilst this variant is common in post-mitotic tissue, its prevalence may be lower in peripheral blood (Zabihi Diba et al., 2016), and so age-related associations may be harder to detect (Mohamed et al., 2006). Despite this, studies examining this deletion (assayed in blood) and cardiometabolic risk factors exist (Botto et al., 2005), but larger, well-powered studies are warranted.

In one of the largest studies of its kind, we have found little evidence that mtDNA CN has an important association with cardiometabolic risk factors in European origin women. An association with insulin in just one of our two cohorts has some consistency with published literature, but may have been a chance finding. Further work should seek to explore whether there is any evidence for an association (and causal effect) of mtDNA CN with insulin.

• The following are the supplementary data related to this article.

Online Resource 1 Literature search terms for PubMed. Online Resource 1

Online Resource 2 Summary of literature examining the relationship between mtDNA CN and various clinical phenotypes. Online Resource 2

Online Resource 3 References for Online Resource S2. Online Resource 3

Online Resource 4 Details of mtDNA CN assay. Online Resource 4

Online Resource 5 Regression analyses for ALSPAC mothers, separately for those with mtDNA CN extracted from a) white cells and b) whole blood. Online Resource 5

Online Resource 6 Fixed- and random-effects meta-analyses of standardised regression of cardiovascular traits on mtDNA CN, for a) All ALSPAC mothers and BWHHS (model M3), and b) restricted to ALSPAC mothers with DNA extracted from whole blood. Online Resource 6
