# Supplementary material for: Cardiometabolic phenotypes and mitochondrial DNA copy number in two cohorts of UK women
Source: Mitochondrion. 2018 Mar;39:9–19. doi: 10.1016/j.mito.2017.08.007 (PMC5832987; doi:10.1016/j.mito.2017.08.007)
Supplement: Online Resource 1 — Literature search terms for PubMed. [file mmc1.pdf]

Title: Cardiometabolic Phenotypes and Mitochondrial DNA Copy Number in Two Cohorts of UK Women
Journal: Mitochondrion

Authors: Anna L Guyatt, Kimberley L Burrows, Philip A I Guthrie, Sue Ring, Wendy McArdle, Ian N M Day, Raimondo Ascione, Debbie A Lawlor, Tom R Gaunt, Santiago Rodriguez

Corresponding author: santi.rodriguez@bristol.ac.uk

## Online Resource 1: Literature search terms for PubMed

("DNA, Mitochondrial"[Mesh:noexp]OR mtDNA[all fields] OR Mitochondrial Deoxyribonucleic Acid[all fields] OR mtDeoxyribonucleic Acid[all fields] or mt Deoxyribonucleic Acid[all fields] OR mt dna[all fields] OR mitochondrial dna[all fields]) AND (copy number*[all fields] OR copynumber*[all fields] OR copy-number*[all fields] OR copy number v*[all fields] OR "DNA copy number variations"[Mesh] OR CNV*[all fields] OR "Gene Dosage"[Mesh] OR copy products[all fields] OR copy quant*[all fields] OR copy reading*[all fields] OR copy var*[all fields] OR copy ratio*[all fields])

AND

(cholesterol[MeSH] or triglycerides[MeSH] or Adiponectin[MeSH] or Leptin[MeSH] or adiponectin[tiab] or leptin[tiab]

or

Blood Cell Count[MeSH] or platelet*[tiab] or lymphocyt*[tiab] or leucocyt*[tiab] or granulocyt*[tiab] or reticulocyt*[tiab] or macrophag*[tiab] or red blood[tiab] or red cell[tiab] or neutrophil[tiab] or basophil[tiab] or eosinophil[tiab] or monocyt*[tiab] or haemoglob*[tiab] or hemoglob*[tiab] or haematocrit*[tiab] or hematocrit*[tiab] or white cell[tiab] or white blood[tiab] or erythrocyt*[tiab]

or

anthropometry[MeSH] or height[tiab] or weight[tiab] or BMI[tiab] or body mass index[tiab] or fatness[tiab] or waist circ*[tiab] or hip circ*[tiab] or whr[tiab] or waist hip ratio[tiab] or waist-hip ratio[tiab] or skinfold*[tiab] or fat mass[tiab] or adipos*[tiab]

Authors: Anna L Guyatt, Kimberley L Burrows, Philip A I Guthrie, Sue Ring, Wendy McArdle, Ian N M Day, Raimondo Ascione, Debbie A Lawlor, Tom R Gaunt, Santiago Rodriguez

Corresponding author: santi.rodriguez@bristol.ac.uk

or

blood pressure[MeSH] or hypertension[MeSH] or systolic[tiab] or diastolic[tiab] or blood pressure[tiab] or hypertens*[tiab]

or

blood glucose[MeSH] or insulin[MeSH] or Diabetes Mellitus[MeSH] or glucose[tiab] or insulin[tiab] or HOMA[tiab] or diabet*[tiab] or T1D[tiab] or T2D[tiab]

or

Interleukin-6[MeSH] or C-Reactive Protein[MeSH] or il-6[tiab] or il6[tiab] or interleukin 6[tiab] or interleukin-6[tiab] or crp[tiab] or c-reactive protein[tiab] or c reactive protein[tiab]

or

smoking[MeSH] or smok*[tiab] or cigar*[tiab]

or

social class[MeSH] or socioeconomic[tiab] or sociodemographic[tiab] or socio-economic[tiab] or socio-demographic[tiab] or social class[tiab] or education[tiab]

or

Cardiovascular Diseases[MeSH] or myocardial inf*[tiab] or heart attack[tiab] or angina[tiab] or stroke[tiab] or transient isch*[tiab] or TIA[tiab] or cerebrovasc*[tiab] or cerebral inf*[tiab])

Filters activated: Humans

Title: Cardiometabolic Phenotypes and Mitochondrial DNA Copy Number in Two Cohorts of UK Women
Journal: Mitochondrion

Authors: Anna L Guyatt, Kimberley L Burrows, Philip A I Guthrie, Sue Ring, Wendy McArdle, Ian N M Day, Raimondo Ascione, Debbie A Lawlor, Tom R Gaunt, Santiago Rodriguez

Corresponding author: santi.rodriguez@bristol.ac.uk

192 papers (Date of search: 02/07/2015); 208 papers (Date of search: 27/10/2016)

Other papers added as necessary.
