# Supplementary material for: Cardiometabolic phenotypes and mitochondrial DNA copy number in two cohorts of UK women
Source: Mitochondrion. 2018 Mar;39:9–19. doi: 10.1016/j.mito.2017.08.007 (PMC5832987; doi:10.1016/j.mito.2017.08.007)
Supplement: Online Resource 2 — Summary of literature examining the relationship between mtDNA CN and various clinical phenotypes. [file mmc2.pdf]

Title: Cardiometabolic Phenotypes and Mitochondrial DNA Copy Number in Two Cohorts of UK Women

Journal: Mitochondrion

Authors: Anna L Guyatt, Kimberley L Burrows, Philip A I Guthrie, Sue Ring, Wendy McArdle, Ian N M Day, Raimondo Ascione, Debbie A Lawlor, Tom R Gaunt, Santiago Rodriguez
Corresponding author: santi.rodriguez@bristol.ac.uk

First author	Year	Domain	N	Outcome(s)	Exposure	Summary	Tissue	Study type
Hernandez-Rios	2013	Dietary	36	MtDNA	Salt intake in obese	Increased MtDNA in salt exposure group	Blood	RCT
Kaaman	2007	Anthropometry	148	MtDNA Lipogenesis	BMI	Decreased with increasing BMI	WAT	XS
					MtDNA	Positive relationship with lipogenesis		
Lee*	2014	Anthropometry	94	MtDNA	Visceral fat area	Visceral fat associated with higher MtDNA	Blood	XS
Ding*	2015	Anthropometry	2077	Weight	MtDNA	No association	Blood	XS
				BMI	MtDNA	No association		
				Waist-hip ratio	MtDNA	Increased MtDNA decreased waist-hip ratio		
				Waist circ.	MtDNA	Increased MtDNA decreased waist circ.		
Lindinger*	2010	Anthropometry	75	MtDNA	BMI	Increased MtDNA count in obesity	OAT	XS
Kim	2012	Anthropometry	44	Telomere length	MtDNA	Correlated, but disappears on adjustment	Blood	XS
Shen	2008	Cell counts	80	NA	NA	Inverse correlation between WBCs/MtDNA	Blood	XS
Urata	2008	Cell counts	NS	MtDNA	Platelets	Platelets contaminate - no nDNA but ++mt	Blood	XS
Pyle*	2010	Cell counts	247	MtDNA	Cell counts	Lympho-, mono-, granulocytes +ve correlation	Blood	XS
Chang	2015	Cognition	110	MtDNA	Depression	MtDNA lower in depression	Blood	XS
Coskun	2012	Cognition	NA	NA	NA	REVIEW: altered in PD, Alzh., Down's Syndrome	NA	Review
Coskun	2004	Cognition	NS	Alzheimers	MtDNA	MtDNA lower in Alzheimers	Brain	XS
Coskun	2010	Cognition	NS	Alzheimers	MtDNA	MtDNA lower in Alzheimers and Downs Syndrome	Brain/Blood	XS
				Downs Syndrome				
De Sousa	2014	Cognition	47	MtDNA	Lithium Rx (BD)	MtDNA lower (trend) in T1BD c.f. controls/T2BD	Blood	XS/RCT
						Lithium treatment no effect.		
Gatt	2013	Cognition	276	PD dementia, LBD	MtDNA	MtDNA lower in prefrontal cortex in PD dementia	Brain	XS
Kim	2011	Cognition	142	Depression	MtDNA	MtDNA lower in depression	Blood	XS
Lee	2010	Cognition	107	NS	NS	MtDNA higher with higher MMSE score	Blood	XS
Podlesniy	2013	Cognition	NS	NS	NS	Risk of Alzheimers correlated with MtDNA	CSF	XS
Rice	2014	Cognition	NS	NS	NS	Lower MtDNA in Alzheimers	Brain	XS
Bersani	2016	PTSD	87	MtDNA	PTSD	Lower MtDNA in PTSD	Blood	XS
Antonetti	1995	Diabetes	NS	NS	NA	Lower MtDNA in DM	SkM	XS
Asmann	2006	Diabetes	30	NA	Hyperglycaemia	IR may lead to muscle mt dysfunction	SkM	XS
					Hyperinsulinaemia			
Chien	2012	Diabetes	43	NS	NS	Lower MtDNA in DM	Vessel?	XS
Hsieh	2011	Diabetes	24	MtDNA	Diabetes	Lower MtDNA in blood but higher in muscle	SkM, Vessel, Blood	XS
Lindinger*	2010	Diabetes	75	MtDNA	Diabetes	No change in DM	OAT	XS
Malik	2009	Diabetes	62	NS	NS	Increased MtDNA in DM	Blood	XS
Rolo	2006	Diabetes	NA	NA	NA	Review? Supports lower MtDNA and DM	NA	Review
Weng	2009	Diabetes	296	MtDNA	Diabetes	Hyperglycaemia, not IR associated with MtDNA	Blood	XS
Bonomi	2012	Hormonal	197	POF	MtDNA	Lower MtDNA in premature ovarian ageing	Blood	XS
Lee	2011	Hormonal	110	PCOS	MtDNA	Lower MtDNA in PCOS	Blood	XS
Ronkainen	2010	Hormonal	22	NS	NS	Muscle MtDNA similar in users/non-users of HRT	SkM	XS
Knez	2016	Hormonal	689	HRT	mtDNA	MtDNA lower in users of estroprogestogens	Blood	XS
Liu	2005	Lipids	154	NS	NS	Decreased MtDNA increased lipids (only in healthy)	Blood	XS
Lee*	2014	Lipids	94	MtDNA	HDL cholesterol	Increased MtDNA count with HDL	Blood	XS
Ding*	2015	Lipids	2077	HDL	MtDNA	No association	Blood	XS
				LDL	MtDNA	No association		
Huang	2011	MetS	130	NS	NS	MtDNA lower in metabolic syndrome	Blood	XS
Kim	2012	MetS	144	Metabolic syndrome	MtDNA	MtDNA lower in metabolic syndrome	Blood	XS
Mozhei	2014	MetS	NS	NS	NS	Tendency to higher BMI lower MtDNA	Blood	XS
Miller	2009	CVD	28	Post-ischaemia recovery	MtDNA	No relationship	Cardiac	XS
Chen	2014	CVD	756	CVD mortality	MtDNA	Lower MtDNA more CVD	Blood	Longitudinal
Ashar	2014	Ageing	16401	All-Cause Mortality	MtDNA	Low MtDNA increased mortality and frailty	Array	XS
				Frailty				
Jylhava	2013	Ageing	174	NS	NS	Ageing/less physical capability lower MtDNA	Blood	XS
Kim	2013	Ageing	129	MtDNA	Telomere length	Positive relationship	Blood	XS
Mengel-From	2014	Ageing	1067	MtDNA	Ageing	Lower MtDNA poorer mortality frailty	Blood	XS/Longitudinal
Huang	2016	Ageing	3400	MtDNA	CVD mortality	Lower MtDNA poorer mortality	Blood	Longitudinal
Qiu	2015	Ageing	75	MtDNA	Telomere length	Positive relationship in pregnancy	Blood	XS
Kim	2012	Bone	146	BMD	MtDNA	Lower MtDNA associated with osteopaenia	Blood	XS
Kim	2014	OSA	40	NS	NS	Lower MtDNA associated with OSA	Blood	XS
Nakahira	2013	Acute mortality	443	Mortality	MtDNA	Higher MtDNA associated with death	Blood	Longitudinal
Pyle*	2010	Acute mortality	247	MtDNA	Apache score	Lower MtDNA with sepsis	Blood	XS
Cote	2007	Acute mortality	28	Mortality	MtDNA	MtDNA lower (non-survivors), higher (survivors).	Blood	Longitudinal
Pavanello	2013	Oxidative stress	90	MtDNA	PAH exposure	Higher MtDNA with exposure	Blood	XS
Qiu	2013	Oxidative stress	40	MtDNA	ox. DNA damage	More oxidative stress more MtDNA	Placenta	XS
Liu	2015	COPD	163	mtDNA	COPD	Lower MtDNA increases COPD risk	Blood	XS

Online Resource 2: *=features more than once. Summary of literature examining the relationship between MtDNA content and various clinical phenotypes. The 'Outcome(s)' and Exposure columns are (mostly) notional only - few studies have undertaken longitudinal assessments. However, most papers include multiple regression analyses. In these cases, the outcome is taken to be the 'y' variable, and the exposure as the primary 'x' variable. Some studies only computed correlations, yet make a hypothesis within the work about the direction of the relationship. When this is unclear, the outcome and exposure variables of studies are annotated as 'Not Stated (NS)'. Abbreviations: MetS=metabolic syndrome; PD=Parkinson's disease; LBD=Lewy Body Dementia; BMD=Bone mineral density; OSA=obstructive sleep apnoea; Premature ovarian failure/poor responders to ovarian hyperstimulation; Lithium RX (BD)= Lithium treatment in bipolar disorder; TIBD=T1 bipolar disoder; T2BD=T2 bipolar disorder; ox. DNA damage = markers of oxidative DNA damage; Alzh.=Alzheimer's disease; IR=insulin resistance; HRT=hormone replacement therapy; WAT/OAT=white, omental adipose tissue; SkM=skeletal muscle; vessel=blood vessel; COPD=Chronic Obstructive pulmonary disease; CVD=cardiovascular disease.
