# Supplementary material for: Cardiometabolic phenotypes and mitochondrial DNA copy number in two cohorts of UK women
Source: Mitochondrion. 2018 Mar;39:9–19. doi: 10.1016/j.mito.2017.08.007 (PMC5832987; doi:10.1016/j.mito.2017.08.007)
Supplement: Online Resource 3 — References for Online Resource S2. [file mmc3.pdf]

Title: Cardiometabolic Phenotypes and Mitochondrial DNA Copy Number in Two Cohorts of UK Women
Journal: Mitochondrion

Authors: Anna L Guyatt, Kimberley L Burrows, Philip A I Guthrie, Sue Ring, Wendy McArdle, Ian N M Day, Raimondo Ascione, Debbie A Lawlor, Tom R Gaunt, Santiago Rodriguez

Corresponding author: santi.rodriguez@bristol.ac.uk; +44 117 3 310 133

## Online Resource 3: References for Online Resource 2[1–50]


1. Hernandez-Rios, R. *et al.* Low fructose and low salt diets increase mitochondrial DNA in white blood cells of overweight subjects. *Exp Clin Endocrinol Diabetes* **121,** 535–538 (2013).

2. Kaaman, M. *et al.* Strong association between mitochondrial DNA copy number and lipogenesis in human white adipose tissue. *Diabetologia* **50,** 2526–2533 (2007).

3. Lee, J. Y., Lee, D. C., Im, J. A. & Lee, J. W. Mitochondrial DNA copy number in peripheral blood is independently associated with visceral fat accumulation in healthy young adults. *Int J Endocrinol* **2014,** 586017 (2014).

4. Lindinger, A. *et al.* Mitochondrial DNA content in human omental adipose tissue. *Obes Surg* **20,** 84–92 (2010).

5. Kim, J. H., Ko, J. H., Lee, D. C., Lim, I. & Bang, H. Habitual physical exercise has beneficial effects on telomere length in postmenopausal women. *Menopause* **19,** 1109–1115 (2012).

6. Shen, M. *et al.* Association between mitochondrial DNA copy number, blood cell counts, and occupational benzene exposure. *Env. Mol Mutagen* **49,** 453–457 (2008).

7. Urata, M., Koga-Wada, Y., Kayamori, Y. & Kang, D. Platelet contamination causes large variation as well as overestimation of mitochondrial DNA content of peripheral blood mononuclear cells. *Ann Clin Biochem* **45,** 513–514 (2008).

8. Pyle, A. *et al.* Fall in circulating mononuclear cell mitochondrial DNA content in human sepsis. *Intensive Care Med* **36,** 956–962 (2010).

9. Chang, C. C., Jou, S. H., Lin, T. T., Lai, T. J. & Liu, C. S. Mitochondria DNA change and oxidative damage in clinically stable patients with major depressive disorder. *PLoS One* **10,** e0125855 (2015).

10. Coskun, P. *et al.* A mitochondrial etiology of Alzheimer and Parkinson disease. *Biochim Biophys Acta* **1820,** 553–564 (2012).

11. Coskun, P. E., Beal, M. F. & Wallace, D. C. Alzheimer's brains harbor somatic mtDNA control-region mutations that suppress mitochondrial transcription and replication. *Proc Natl Acad Sci U S A* **101,** 10726–10731 (2004).

12. Coskun, P. E. *et al.* Systemic mitochondrial dysfunction and the etiology of Alzheimer's disease and down syndrome dementia. *J Alzheimers Dis* **20 Suppl 2,** S293–310 (2010).

Authors: Anna L Guyatt, Kimberley L Burrows, Philip A I Guthrie, Sue Ring, Wendy McArdle, Ian N M Day, Raimondo Ascione, Debbie A Lawlor, Tom R Gaunt, Santiago Rodriguez

Corresponding author: santi.rodriguez@bristol.ac.uk; +44 117 3 310 133

13. De Sousa, R. T. *et al.* Leukocyte mitochondrial DNA copy number in bipolar disorder. *Prog Neuropsychopharmacol Biol Psychiatry* **48,** 32–35 (2014).

14. Gatt, A. P., Jones, E. L., Francis, P. T., Ballard, C. & Bateman, J. M. Association of a polymorphism in mitochondrial transcription factor A (TFAM) with Parkinson’s disease dementia but not dementia with Lewy bodies. *Neurosci Lett* **557 Pt B,** 177–180 (2013).

15. Kim, M. Y., Lee, J. W., Kang, H. C., Kim, E. & Lee, D. C. Leukocyte mitochondrial DNA (mtDNA) content is associated with depression in old women. *Arch Gerontol Geriatr* **53,** e218–21 (2011).

16. Lee, J. W., Park, K. D., Im, J. A., Kim, M. Y. & Lee, D. C. Mitochondrial DNA copy number in peripheral blood is associated with cognitive function in apparently healthy elderly women. *Clin Chim Acta* **411,** 592–596 (2010).

17. Podlesniy, P. *et al.* Low cerebrospinal fluid concentration of mitochondrial DNA in preclinical Alzheimer disease. *Ann Neurol* **74,** 655–668 (2013).

18. Rice, A. C. *et al.* Mitochondrial DNA copy numbers in pyramidal neurons are decreased and mitochondrial biogenesis transcriptome signaling is disrupted in Alzheimer’s disease hippocampi. *J Alzheimers Dis* **40,** 319–330 (2014).

19. Bersani, F. S. *et al.* Mitochondrial DNA copy number is reduced in male combat veterans with PTSD. *Prog. Neuropsychopharmacol. Biol. Psychiatry* **64,** 10–7 (2016).

20. Antonetti, D. A., Reynet, C. & Kahn, C. R. Increased expression of mitochondrial-encoded genes in skeletal muscle of humans with diabetes mellitus. *J Clin Invest* **95,** 1383–1388 (1995).

21. Asmann, Y. W. *et al.* Skeletal muscle mitochondrial functions, mitochondrial DNA copy numbers, and gene transcript profiles in type 2 diabetic and nondiabetic subjects at equal levels of low or high insulin and euglycemia. *Diabetes* **55,** 3309–3319 (2006).

22. Chien, M. C. *et al.* Role of mitochondrial DNA variants and copy number in diabetic atherogenesis. *Genet Mol Res* **11,** 3339–3348 (2012).

23. Hsieh, C. J. *et al.* Tissue-specific differences in mitochondrial DNA content in type 2 diabetes. *Diabetes Res Clin Pr.* **92,** 106–110 (2011).

24. Malik, A. N., Shahni, R. & Iqbal, M. M. Increased peripheral blood mitochondrial DNA in type 2 diabetic patients with nephropathy. *Diabetes Res Clin Pr.* **86,** e22–4 (2009).

Authors: Anna L Guyatt, Kimberley L Burrows, Philip A I Guthrie, Sue Ring, Wendy McArdle, Ian N M Day, Raimondo Ascione, Debbie A Lawlor, Tom R Gaunt, Santiago Rodriguez

Corresponding author: santi.rodriguez@bristol.ac.uk; +44 117 3 310 133

25. Rolo, A. P. & Palmeira, C. M. Diabetes and mitochondrial function: role of hyperglycemia and oxidative stress. *Toxicol Appl Pharmacol* **212,** 167–178 (2006).

26. Weng, S. W. *et al.* Peripheral blood mitochondrial DNA content and dysregulation of glucose metabolism. *Diabetes Res Clin Pr.* **83,** 94–99 (2009).

27. Bonomi, M. *et al.* Blood cell mitochondrial DNA content and premature ovarian aging. *PLoS One* **7,** e42423 (2012).

28. Lee, S. H. *et al.* Mitochondrial DNA copy number in peripheral blood in polycystic ovary syndrome. *Metabolism* **60,** 1677–1682 (2011).

29. Ronkainen, P. H. *et al.* Global gene expression profiles in skeletal muscle of monozygotic female twins discordant for hormone replacement therapy. *Aging Cell* **9,** 1098–1110 (2010).

30. Knez, J. *et al.* Correlates of Peripheral Blood Mitochondrial DNA Content in a General Population. *Am. J. Epidemiol.* **183,** 138–46 (2016).

31. Liu, C. S. *et al.* Alteration of the copy number of mitochondrial DNA in leukocytes of patients with hyperlipidemia. *Ann N Y Acad Sci* **1042,** 70–75 (2005).

32. Ding, J. *et al.* Assessing Mitochondrial DNA Variation and Copy Number in Lymphocytes of ~2,000 Sardinians Using Tailored Sequencing Analysis Tools. *PLoS Genet.* **11,** e1005306 (2015).

33. Huang, C. H. *et al.* Depleted leukocyte mitochondrial DNA copy number in metabolic syndrome. *J Atheroscler Thromb* **18,** 867–873 (2011).

34. Kim, J. H., Im, J. A. & Lee, D. C. The relationship between leukocyte mitochondrial DNA contents and metabolic syndrome in postmenopausal women. *Menopause* **19,** 582–587 (2012).

35. Mozhei, O. I. *et al.* [Evaluating the mitochondrial dna copy number in leukocytes and adipocytes from metabolic syndrome patients: pilot study]. *Mol Biol* **48,** 677–681 (2014).

36. Miller, F. *et al.* Age-related decline in stress responses of human myocardium may not be explained by changes in mtDNA. *Mech Ageing Dev* **130,** 742–747 (2009).

37. Chen, S. *et al.* Association between leukocyte mitochondrial DNA content and risk of coronary heart disease: a case-control study. *Atherosclerosis* **237,** 220–226 (2014).

Authors: Anna L Guyatt, Kimberley L Burrows, Philip A I Guthrie, Sue Ring, Wendy McArdle, Ian N M Day, Raimondo Ascione, Debbie A Lawlor, Tom R Gaunt, Santiago Rodriguez

Corresponding author: santi.rodriguez@bristol.ac.uk; +44 117 3 310 133

38. Ashar, F. N. *et al.* Association of mitochondrial DNA levels with frailty and all-cause mortality. *J Mol Med* **93,** 177–186 (2015).

39. Jylhava, J. *et al.* Characterization of the role of distinct plasma cell-free DNA species in age-associated inflammation and frailty. *Aging Cell* **12,** 388–397 (2013).

40. Kim, J. H., Kim, H. K., Ko, J. H., Bang, H. & Lee, D. C. The relationship between leukocyte mitochondrial DNA copy number and telomere length in community-dwelling elderly women. *PLoS ONE [Electronic Resour.* **8,** e67227 (2013).

41. Mengel-From, J. *et al.* Mitochondrial DNA copy number in peripheral blood cells declines with age and is associated with general health among elderly. *Hum Genet* **133,** 1149–1159 (2014).

42. Huang, J. *et al.* Decreased Peripheral Mitochondrial DNA Copy Number is Associated with the Risk of Heart Failure and Long-term Outcomes. *Medicine (Baltimore).* **95,** e3323 (2016).

43. Qiu, C., Enquobahrie, D. A., Gelaye, B., Hevner, K. & Williams, M. A. The association between leukocyte telomere length and mitochondrial DNA copy number in pregnant women: a pilot study. *Clin Lab* **61,** 363–369 (2015).

44. Kim, J. H. & Lee, D. C. Mitochondrial DNA copy number in peripheral blood is associated with femoral neck bone mineral density in postmenopausal women. *J Rheumatol* **39,** 1465–1472 (2012).

45. Kim, Y. S. *et al.* Can mitochondrial dysfunction be a predictive factor for oxidative stress in patients with obstructive sleep apnea? *Antioxid Redox Signal* **21,** 1285–1288 (2014).

46. Nakahira, K. *et al.* Circulating mitochondrial DNA in patients in the ICU as a marker of mortality: derivation and validation. *PLoS Med* **10,** e1001577; discussion e1001577 (2013).

47. Cote, H. C., Day, A. G. & Heyland, D. K. Longitudinal increases in mitochondrial DNA levels in blood cells are associated with survival in critically ill patients. *Crit Care* **11,** R88 (2007).

48. Pavanello, S. *et al.* Mitochondrial DNA copy number and exposure to polycyclic aromatic hydrocarbons. *Cancer Epidemiol Biomarkers Prev* **22,** 1722–1729 (2013).

49. Qiu, C. *et al.* Mitochondrial DNA copy number and oxidative DNA damage in placental tissues from gestational diabetes and control pregnancies: a pilot study. *Clin Lab* **59,** 655–660 (2013).

Authors: Anna L Guyatt, Kimberley L Burrows, Philip A I Guthrie, Sue Ring, Wendy McArdle, Ian N M Day, Raimondo Ascione, Debbie A Lawlor, Tom R Gaunt, Santiago Rodriguez

Corresponding author: santi.rodriguez@bristol.ac.uk; +44 117 3 310 133

50. Liu, S.-F. *et al.* Leukocyte Mitochondrial DNA Copy Number Is Associated with Chronic Obstructive Pulmonary Disease. *PLoS One* **10,** e0138716 (2015).
