# Supplementary material for: Cardiometabolic phenotypes and mitochondrial DNA copy number in two cohorts of UK women
Source: Mitochondrion. 2018 Mar;39:9–19. doi: 10.1016/j.mito.2017.08.007 (PMC5832987; doi:10.1016/j.mito.2017.08.007)
Supplement: Online Resource 4 — Details of mtDNA CN assay. [file mmc4.pdf]

Title: Cardiometabolic Phenotypes and Mitochondrial DNA Copy Number in Two Cohorts of UK Women
Journal: Mitochondrion

Authors: Anna L Guyatt, Kimberley L Burrows, Philip A I Guthrie, Sue Ring, Wendy McArdle, Ian N M Day, Raimondo Ascione, Debbie A Lawlor, Tom R Gaunt, Santiago Rodriguez

Corresponding author: santi.rodriguez@bristol.ac.uk; +44 117 3 310 133

Online Resource 4: Mitochondrial DNA copy number assay details

For each of the mitochondrial and nuclear gene reactions, a master mix was made using 5µL SensiFAST SYBR No-Rox Kit (Bioline), 0.50µL water, and 250nM of each of the forward and reverse primers. Primer sequences (5'-3') were hmitoF5 CTTCTGGCCACAGCACTTAAAC and hmitoR5 GCTGGTGTTAGGGTTCTTTGTTTT for the MtDNA amplicon, and hB2M_F2 GCTGGGTAGCTCTAAACAATGTATTCA and hB2M_R2 CCATGTACTAACAAATGTCTAAAATGGT for the nuclear DNA amplicon (https://www.ncbi.nlm.nih.gov/pubmed/21703239). DNA was diluted to a working concentration of 0.25ng/µL, and 4µL (i.e. 1ng) of DNA was added to 6 microplate wells, to which 6µL aliquots of the respective master mixes were added, giving a final reaction volume of 10µL per well. Each of the mitochondrial and nuclear reactions was undertaken in triplicate. Samples were assayed using a Roche LightCycler LC480 and 384-well plates under the following thermocycler conditions: 5 minutes at 95^{0}C (1 cycle), then 45 cycles of: 5 seconds at 95^{0}C→15 seconds at 55^{0}C → 15 seconds at 72^{0}C → 1 second at 78^{0}C (with signal acquisition).

Standard curves were generated from the pooled results of 4 different DNAs replicated in 3 blocks, each block containing 4 wells of DNA for mtDNA primers and 4 wells of DNA for nuDNA primers. PCR efficiency values were calculated from the standard curves by LC480 software and used to adjust raw Cp data.

MtDNA copy number was calculated as the relative magnitude of the signal from the mitochondrial amplicon to the nuclear amplicon, using the following equation:

$$mtDNA\ copy\ number = 2^{(nuclear\ DNA\ Cp - mitochondrial\ DNA\ Cp)}$$

Authors: Anna L Guyatt, Kimberley L Burrows, Philip A I Guthrie, Sue Ring, Wendy McArdle, Ian N M Day, Raimondo Ascione, Debbie A Lawlor, Tom R Gaunt, Santiago Rodriguez

Corresponding author: santi.rodriguez@bristol.ac.uk; +44 117 3 310 133

i.e. the mitochondrial DNA copy number was calculated from the mitochondrial DNA signal relative to the single-copy nuclear DNA signal. To take account of plate-to-plate variability, 3 lots of pooled, aliquotted and frozen 'calibrator' DNA were amplified on each microplate, along with a negative (no DNA) control. The median calibrator value was obtained for every microplate, and an average of these across all microplates was calculated. This average calibrator value was then divided by the median calibrator value for each microplate, to generate a calibration factor for that microplate which was applied to the previously calculated copy numbers; the result was the final efficiency- and calibrator- adjusted value for mtDNA copy number for each microplate.
