# Supplementary material for: Cardiometabolic phenotypes and mitochondrial DNA copy number in two cohorts of UK women
Source: Mitochondrion. 2018 Mar;39:9–19. doi: 10.1016/j.mito.2017.08.007 (PMC5832987; doi:10.1016/j.mito.2017.08.007)
Supplement: Online Resource 5 — Regression analyses for ALSPAC mothers, separately for those with mtDNA CN extracted from a) white cells and b) whole blood. [file mmc5.pdf]

Outcome	M1					M2					M3				
	B	LCI	UCI	P	N	B	LCI	UCI	P	N	B	LCI	UCI	P	N
Cholesterol*	0.043	-0.010	0.096	0.110	1228	0.037	-0.016	0.090	0.169	1228	0.113	-0.023	0.249	0.105	251
HDL*	0.026	-0.027	0.080	0.335	1228	0.013	-0.041	0.067	0.632	1228	0.007	-0.130	0.144	0.919	251
LDL*	0.020	-0.034	0.073	0.471	1228	0.018	-0.036	0.072	0.505	1228	0.068	-0.069	0.205	0.331	251
Triglycerides*	0.035	-0.019	0.089	0.207	1228	0.036	-0.019	0.091	0.204	1228	0.126	-0.023	0.275	0.099	251
Glucose*	0.005	-0.048	0.058	0.847	1228	0.008	-0.047	0.062	0.781	1228	0.078	-0.060	0.215	0.271	251
Insulin*	0.000	-0.054	0.054	0.997	1224	0.013	-0.042	0.068	0.638	1224	0.048	-0.085	0.180	0.480	250
C-reactive protein*	-0.010	-0.063	0.044	0.721	1228	-0.002	-0.056	0.053	0.952	1228	-0.075	-0.220	0.069	0.309	251
Body Mass Index*	-0.016	-0.066	0.035	0.551	1369	-0.003	-0.054	0.049	0.918	1369	-0.014	-0.150	0.122	0.841	261
Waist-Hip Ratio	0.035	-0.016	0.085	0.176	1371	0.048	-0.003	0.099	0.067	1371	0.004	-0.128	0.136	0.956	262
Height	-0.023	-0.074	0.028	0.368	1372	-0.017	-0.069	0.035	0.532	1372	-0.036	-0.171	0.100	0.607	262
Systolic BP	0.023	-0.028	0.074	0.383	1338	0.028	-0.024	0.080	0.296	1338	0.005	-0.130	0.139	0.946	256
Diastolic BP	-0.004	-0.055	0.047	0.872	1338	0.001	-0.051	0.054	0.970	1338	-0.062	-0.195	0.071	0.360	256

Online Resource 5a: Standardised linear regression of cardiovascular traits on mtDNA copy number (ALSPAC, white cell DNA samples only). Abbreviations: M1=Model 1 (unadjusted); M2=Model 2 (adjusted for age at DNA sampling, DNA source, education level, smoking status, occupational class, DNA concentration); M3=Model 3 (as M2, plus adjustment for cell counts as described in Table 2b). B=Standardised beta coefficient; LCI=95 percent confidence interval (lower bound); UCI=95 percent confidence interval (upper bound); HDL, LDL=High-, Low-density lipoprotein cholesterol. BP=blood pressure. *=log-transformed.

Title: Cardiometabolic Phenotypes and Mitochondrial DNA Copy Number in Two Cohorts of UK Women
Journal: Mitochondrion
Authors: Anna L Guyatt, Kimberley L Burrows, Philip A I Guthrie, Sue Ring, Wendy McArdle, Ian N M Day, Raimondo Ascione, Debbie A Lawlor, Tom R Gaunt, Santiago Rodriguez
Corresponding author: santi.rodriguez@bristol.ac.uk

Outcome	M1					M2					M3				
	B	LCI	UCI	P	N	B	LCI	UCI	P	N	B	LCI	UCI	P	N
Cholesterol*	0.126	0.036	0.215	0.006	822	0.113	0.024	0.201	0.013	822	0.037	-0.151	0.225	0.699	214
HDL*	0.016	-0.072	0.104	0.721	822	0.013	-0.076	0.101	0.780	822	-0.002	-0.179	0.174	0.978	214
LDL*	0.099	0.010	0.188	0.029	822	0.087	-0.003	0.176	0.059	822	0.018	-0.171	0.208	0.849	214
Triglycerides*	0.083	-0.004	0.169	0.062	822	0.082	-0.006	0.169	0.068	822	0.002	-0.171	0.176	0.978	214
Glucose*	0.062	-0.028	0.152	0.177	822	0.066	-0.026	0.158	0.159	822	0.042	-0.138	0.222	0.648	214
Insulin*	0.044	-0.044	0.132	0.330	820	0.055	-0.035	0.145	0.230	820	0.017	-0.181	0.215	0.866	214
C-reactive protein*	0.062	-0.026	0.150	0.167	822	0.058	-0.030	0.147	0.199	822	-0.022	-0.209	0.165	0.821	214
Body Mass Index*	0.003	-0.080	0.086	0.952	897	0.002	-0.082	0.086	0.958	897	-0.187	-0.374	0.000	0.051	227
Waist-Hip Ratio	0.104	0.019	0.189	0.016	896	0.101	0.016	0.186	0.020	896	-0.035	-0.214	0.144	0.701	227
Height	-0.026	-0.108	0.057	0.546	897	-0.024	-0.108	0.061	0.579	897	-0.069	-0.249	0.110	0.450	227
Systolic BP	0.035	-0.050	0.120	0.421	876	0.026	-0.060	0.113	0.550	876	-0.145	-0.324	0.034	0.114	217
Diastolic BP	0.052	-0.033	0.137	0.234	876	0.055	-0.032	0.142	0.215	876	-0.103	-0.286	0.079	0.267	217

Online Resource 5b: Standardised linear regression of cardiovascular traits on mtDNA copy number (ALSPAC, whole blood DNA samples only). Abbreviations: M1=Model 1 (unadjusted); M2=Model 2 (adjusted for age at DNA sampling, DNA source, education level, smoking status, occupational class, DNA concentration); M3=Model 3 (as M2, plus adjustment for cell counts as described in Table 2b). B=Standardised beta coefficient; LCI=95 percent confidence interval (lower bound); UCI=95 percent confidence interval (upper bound); HDL, LDL=High-, Low-density lipoprotein cholesterol. BP=blood pressure. *=log-transformed.

Title: Cardiometabolic Phenotypes and Mitochondrial DNA Copy Number in Two Cohorts of UK Women

Journal: Mitochondrion

Authors: Anna L Guyatt, Kimberley L Burrows, Philip A I Guthrie, Sue Ring, Wendy McArdle, Ian N M Day, Raimondo Ascione, Debbie A Lawlor, Tom R Gaunt, Santiago Rodriguez
Corresponding author: santi.rodriguez@bristol.ac.uk
