# Supplementary material for: Cardiometabolic phenotypes and mitochondrial DNA copy number in two cohorts of UK women
Source: Mitochondrion. 2018 Mar;39:9–19. doi: 10.1016/j.mito.2017.08.007 (PMC5832987; doi:10.1016/j.mito.2017.08.007)
Supplement: Online Resource 6 — Fixed- and random-effects meta-analyses of standardised regression of cardiovascular traits on mtDNA CN, for a) All ALSPAC mothers and BWHHS (model M3), and b) restricted to ALSPAC mothers with DNA extracted from whole blood. [file mmc6.pdf]

Variable	Fixed						Random					
	B	LCI	UCI	P	N	I^2	B	LCI	UCI	P	N	I^2
Cholesterol*	0.023	-0.013	0.058	0.21600	3289	55	0.041	-0.038	0.119	0.3090	3289	55
HDL*	-0.019	-0.054	0.017	0.29900	3285	0	-0.019	-0.054	0.017	0.2990	3285	0
LDL*	0.030	-0.007	0.066	0.11000	3228	0	0.030	-0.007	0.066	0.1100	3228	0
Triglycerides*	-0.008	-0.043	0.027	0.64300	3289	67	0.019	-0.078	0.116	0.6990	3289	67
Glucose*	-0.015	-0.051	0.021	0.42200	3282	38	-0.004	-0.067	0.058	0.8920	3282	38
Insulin*	-0.051	-0.087	-0.016	0.00493	3293	38	-0.041	-0.104	0.023	0.2110	3293	38
C-reactive protein*	-0.011	-0.046	0.025	0.55600	3115	0	-0.011	-0.046	0.025	0.5560	3115	0
Body Mass Index*	0.003	-0.033	0.039	0.87600	3299	74	-0.030	-0.138	0.079	0.5930	3299	74
Waist-Hip Ratio	-0.019	-0.055	0.016	0.28800	3286	0	-0.019	-0.055	0.016	0.2880	3286	0
Height	-0.030	-0.065	0.005	0.09630	3301	0	-0.030	-0.065	0.005	0.0963	3301	0
Systolic BP	-0.035	-0.069	0.000	0.04990	3303	0	-0.035	-0.069	0.000	0.0499	3303	0
Diastolic BP	-0.044	-0.080	-0.008	0.01560	3303	0	-0.044	-0.080	-0.008	0.0156	3303	0

Online Resource 6a: Fixed- and random-effects meta-analyses of standardised regression of cardiovascular traits on mtDNA copy in ALSPAC and BWHHS (adjusted model M3). B=Standardised beta coefficient; LCI=95 percent confidence interval (lower bound); UCI=95 percent confidence interval (upper bound); HDL, LDL=High-, Low-density lipoprotein cholesterol. BP=blood pressure. I2=I-squared statistic for heterogeneity. *=log-transformed.

Title: Cardiometabolic Phenotypes and Mitochondrial DNA Copy Number in Two Cohorts of UK Women

Journal: Mitochondrion

Authors: Anna L Guyatt, Kimberley L Burrows, Philip A I Guthrie, Sue Ring, Wendy McArdle, Ian N M Day, Raimondo Ascione, Debbie A Lawlor, Tom R Gaunt, Santiago Rodriguez
Corresponding author: santi.rodriguez@bristol.ac.uk

Variable	Fixed						Random					
	B	LCI	UCI	P	N	I^2	B	LCI	UCI	P	N	I^2
Cholesterol*	0.014	-0.024	0.051	0.46900	3039	0	0.014	-0.024	0.051	0.46900	3039	0
HDL*	-0.020	-0.056	0.017	0.29200	3035	0	-0.020	-0.056	0.017	0.29200	3035	0
LDL*	0.024	-0.014	0.062	0.20900	2978	0	0.024	-0.014	0.062	0.20900	2978	0
Triglycerides*	-0.018	-0.054	0.018	0.32900	3039	0	-0.018	-0.054	0.018	0.32900	3039	0
Glucose*	-0.020	-0.058	0.017	0.28600	3032	0	-0.020	-0.058	0.017	0.28600	3032	0
Insulin*	-0.057	-0.094	-0.020	0.00283	3044	0	-0.057	-0.094	-0.020	0.00283	3044	0
C-reactive protein*	-0.006	-0.043	0.031	0.74100	2865	0	-0.006	-0.043	0.031	0.74100	2865	0
Body Mass Index*	0.007	-0.030	0.045	0.69300	3039	77	-0.065	-0.259	0.130	0.51500	3039	77
Waist-Hip Ratio	-0.022	-0.059	0.015	0.24700	3025	0	-0.022	-0.059	0.015	0.24700	3025	0
Height	-0.030	-0.066	0.007	0.11200	3040	0	-0.030	-0.066	0.007	0.11200	3040	0
Systolic BP	-0.038	-0.074	-0.002	0.04090	3048	31	-0.054	-0.139	0.031	0.21600	3048	31
Diastolic BP	-0.044	-0.081	-0.006	0.02230	3048	0	-0.044	-0.081	-0.006	0.02230	3048	0

Online Resource 6b: Fixed- and random-effects meta-analyses of standardised regression of cardiovascular traits on mtDNA copy in ALSPAC (whole blood samples only) and BWHHS (adjusted model M3). B=Standardised beta coefficient; LCI=95 percent confidence interval (lower bound); UCI=95 percent confidence interval (upper bound); HDL, LDL=High-, Low-density lipoprotein cholesterol. BP=blood pressure. I2=I-squared statistic for heterogeneity. *=log-transformed.

Title: Cardiometabolic Phenotypes and Mitochondrial DNA Copy Number in Two Cohorts of UK Women

Journal: Mitochondrion

Authors: Anna L Guyatt, Kimberley L Burrows, Philip A I Guthrie, Sue Ring, Wendy McArdle, Ian N M Day, Raimondo Ascione, Debbie A Lawlor, Tom R Gaunt, Santiago Rodriguez
Corresponding author: santi.rodriguez@bristol.ac.uk
